# A Case of Late Dumping Syndrome in a Post-bariatric Pregnant Lady Seen in a Primary Care Clinic

**DOI:** 10.7759/cureus.34926

**Published:** 2023-02-13

**Authors:** Wong Voon Son, Anu Suria Ganason, Waye Hann Kang

**Affiliations:** 1 Department of Population Medicine, M. Kandiah Faculty of Medicine and Health Sciences, Universiti Tunku Abdul Rahman, Kajang, MYS; 2 Department of Primary Healthcare, Universiti Sains Islam Malaysia, Nilai, MYS; 3 Department of Medicine, M. Kandiah Faculty of Medicine and Health Sciences, Universiti Tunku Abdul Rahman, Kajang, MYS

**Keywords:** nutritional deficiency, hypoglycaemia, pregnancy, bariatric surgery, dumping syndrome

## Abstract

Dumping syndrome is a common complication of bariatric surgery. A high clinical suspicion of hypoglycaemic events is required as the symptoms mimic early pregnancy complaints. Diagnosis and treatment of dumping syndrome remain a challenge in pregnancy. Thus, diet modification remains a mainstay of management. This case report discusses dumping syndrome in a post-bariatric surgery mother who presented hypoglycaemia symptoms in the primary care clinic.

## Introduction

As the number of bariatric surgeries among women of childbearing age increases, dumping syndrome is not infrequently seen in pregnancy [1]. Bariatric surgeries improve anovulation and lead to spontaneous pregnancy [2]. Studies revealed that post-bariatric surgery, women have lower incidences of gestational diabetes and pregnancy-induced hypertension, and better fetal outcomes [3]. However, pregnancy following bariatric surgery poses surgical, medical, and obstetric challenges, which require multidisciplinary team management [4]. Thus, pre-pregnancy care is critical to ensure mother and fetal wellbeing. A pregnant mother with dumping syndrome may present with abdominal pain, nausea, palpitation, or tremor, which can be a physiological manifestation of pregnancy [1]. Primary care physicians should be aware of possible symptoms of dumping syndrome and assess nutritional deficiencies in pregnant mothers following bariatric surgery [5].

## Case presentation

A 37-year-old lady (gravida 2 para 0+1) had a booking visit at her local maternal health clinic during her first trimester. She has had a history of bariatric surgery, given morbid obesity with a body mass index (BMI) of 40, and subfertility with polycystic ovarian syndrome. She underwent bariatric surgery in 2020 but defaulted on her subsequent follow-up. She successfully lost 41 kg and eventually conceived spontaneously 18 months post-surgery.

During her booking visit, she was screened for diabetes mellitus at nine weeks of gestation because of her advanced maternal age and her strong family history of diabetes mellitus. Her oral glucose tolerance test result was 4.2 (fasting) and 10.8 mmol/L (two hours post 75 g of glucose in 200 ml of water). She was then on medical nutrition therapy, with her home self-blood sugar monitoring ranging between 3.7 and 4.8 mmol/L. She reported a few episodes of hypoglycaemic symptoms such as sweating, hunger, and hand tremor, especially at two to three hours postprandial during her second trimester. Her capillary glucose reading documented during these hypoglycaemic events was within 2.5-2.8 mmol/L. She was subsequently diagnosed with late dumping syndrome and referred to the antenatal clinic for combined care. She was advised to reduce carbohydrate loads and space carbohydrate intake with a high protein diet and vegetables. With diet adjustment, hypoglycaemic events resolved.

Her investigation revealed an iron deficiency anaemia with a haemoglobin of 9.8 g/dL. Following booking, the haemoglobin level was 11 g/dl. However, it slowly dropped to 10.2 g/dl and then 9.8 g/dL with hypochromic microcytic features (Table 1). She had a low serum iron, ferritin, and calcium level (Table 2). She was then started on a tablet of calcium carbonate 1 g twice daily, Iberet tablet once daily, and multivitamins. She was later diagnosed as a group B *Streptococcus* carrier at 10 weeks gestation when she presented with per vaginal discharge. Furthermore, at 24 weeks of gestation, she presented with chorioamnionitis symptoms and delivered a baby boy prematurely with a birth weight of 710 g. The baby was admitted to the neonatal intensive care unit and passed away on day four of life due to severe prematurity. Her post-partum period was uneventful, and no hypoglycaemia episodes were noted.

**Table 1:** Serial readings of full blood count

Full blood count				
Gestation age	9 weeks	15 weeks	20 weeks	Normal reading
Haemoglobin (g/dl)	11	↓10.2	↓9.8	13-17
Mean corpuscular haemoglobin (MCH) (pg)	31	29	↓25	27.5-33.2
Mean corpuscular volume (MCV) (fl)	92	91	↓78	80-100
Platelets (PLT) (x10^9^/L)	289	299	215	150-450

**Table 2:** List of blood investigations and results

Blood investigation	Reading	Normal reading
Oral glucose tolerance test		
Fasting blood sugar (mmol/L)	4.2	4-5.1
Two hours postprandial glucose (mmol/L)	↑10.8	4-7.8
Blood sugar monitoring at home		
Fasting capillary glucose (mmol/L)	4.0-4.9	4-5.3
One hour postprandial glucose (mmol/L)	3.7-4.8	4-7.8
Capillary blood sugar during hypoglycaemia event (mmol/L)	↓2.5-2.8	4-7.8
Iron studies		
Iron (umol/L)	↓6.7	10.74-30.43
Ferritin (umol/mL)	↓5.8	18-160
Total iron-binding capacity (umol/L)	↑83.9	42-80.5
Renal profile		
Urea (mg/dl)	1.8	6-24
Sodium (mmol/L)	138	135-145
Potassium (mmol/L)	4.2	3.5-5
Creatinine (mmol/L)	39	95-105
Other blood tests		
Corrected Ca (mmol/L)	↓2.15	2.25-2.65
Phosphate (mg/dl)	1.26	0.85-1.1
Magnesium (mmol/L)	0.75	0.85-1.58
Vitamin D (ng/ml)	31.5	30-100
Folate (nmol/L)	10.48	3-18
Vitamin B12 (pmol/L)	182.8	180-914

## Discussion

Obesity is a worldwide health issue impacting about 650 million individuals, particularly during the reproductive years [6]. In Malaysia, one in two adults is overweight or obese, according to the National Health Morbidity Survey 2019. Obesity was higher in females (up to 54.4%), and one in five mothers was obese when conceived [7]. There is an increase in bariatric surgeries among women of childbearing age to achieve sustained weight loss. Thus, family physicians should be aware of the complications of bariatric surgery and its impact on pregnancies [8]. All active reproductive women who underwent bariatric surgery should be provided pre-pregnancy counselling. The conventional type of contraception should be considered as the efficacy of hormonal contraception may be affected due to malabsorption post-bariatric surgery. Pregnancy should be postponed for 12 to 24 months post-bariatric surgery until weight is stabilized [9]. There is a higher risk of miscarriage, fetal malnutrition, and intrauterine growth retardation if conceived in the post-bariatric period [4,10]. There is some indication, nevertheless, that pregnancy within the first year after surgery may have a negative outcome [11].

The typical nutritional deficiencies found after bariatric surgery are iron, folate, vitamin B1, B12, and D, and calcium. These micronutrients are essential for maternal health and fetal growth. Severe deficiency of micronutrients can cause adverse effects of pregnancy, such as anaemia and congenital abnormality. A multidisciplinary team should therefore be involved in pre-pregnancy care. Mineral supplements and multivitamins should be prescribed before and during pregnancy [8,12].

Dumping syndrome is a common complication of post-bariatric surgery. It has a higher risk of maternal hypoglycaemia and subsequent fetal hypoglycaemia resulting in intrauterine growth restriction and small gestation-age infants [3]. There are few postulated mechanisms towards dumping syndrome. The pathophysiology of dumping syndrome can be multifocal and not well understood. It can be either early dumping or late dumping [4]. During the early gestational period, there is a physiological increase in insulin secretion and insulin hypersensitivity. These subsequently increase the risk of hypoglycaemia in pregnant ladies post-bariatric surgery. In post-bariatric surgery, rapid gastric emptying and glucose absorption following smaller stomach capacity can cause hyperinsulinemia and reactive hypoglycaemia [1].

Early dumping syndrome usually happens 15 minutes to one-hour post-meal. It is due to the rapid transit of gastric content causing osmotic shifts in the proximal small intestine. Vasomotor symptoms develop as a consequence of a decrease in blood pressure [12]. Patients are advised to have smaller meals up to six times per day and avoid fluid intake within 30 minutes after the meal. They should be supine for 30 minutes to reduce vasomotor symptoms by prolonging gastric emptying [6,8].

Late dumping syndrome usually happens one to three hours postprandial. It is further explained in this patient as she has a recurrent episode of hypoglycaemic effects two to three hours postprandial [12]. Dietary modifications include advice on low glycaemic index foods, elimination of simple carbohydrates, and daily protein of 60 g [5]. The oral glucose tolerance test (OGTT) is frequently poorly tolerated and less accurate in women post-bariatric surgery. OGTT should be substituted with home capillary blood sugar measurement over one week in 24 to 28 weeks [1,12]. The patient’s symptoms improved significantly following guided dietary advice. However, in view of nutritional deficiency and group B *Streptococcus* infection, there is a possibility of complications in pregnancy leading to premature delivery.

Women following Roux-en-Y gastric bypass are at higher risk of developing internal hernia during pregnancy [13]. They may present with abdominal pain and vomiting, easily mistaken for pregnancy-related complaints. Immediate surgical intervention must be considered in abdominal pain, regardless of pregnancy. Other surgical complication includes adhesion, band slippage, and small intestine ischemia. The course of labour and delivery should not be affected by a patient's prior history of bariatric surgery [1,5].

## Conclusions

Women are prone to clinical challenges in subsequent pregnancies following bariatric surgery. A physician should provide pre-pregnancy counselling and regular assessment of nutritional status prior to conception. Clinical difficulties can arise following bariatric surgery in pregnant women of reproductive age. Thus, pregnancies that follow bariatric surgery require extensive team involvement due to the high risk of complications.

## References

[1] Narayanan RP, Syed AA (2016). Pregnancy following bariatric surgery—medical complications and management. Obes Surg.

[2] Pg Baharuddin DM, Payus AO, Abdel Malek Fahmy EH (2021). Bariatric surgery and its impact on fertility, pregnancy and its outcome: a narrative review. Ann Med Surg (Lond).

[3] Galazis N, Docheva N, Simillis C, Nicolaides KH (2014). Maternal and neonatal outcomes in women undergoing bariatric surgery: a systematic review and meta-analysis. Eur J Obstet Gynecol Reprod Biol.

[4] Shawe J, Ceulemans D, Akhter Z (2019). Pregnancy after bariatric surgery: consensus recommendations for periconception, antenatal and postnatal care. Obes Rev.

[5] Falcone V, Stopp T, Feichtinger M (2018). Pregnancy after bariatric surgery: a narrative literature review and discussion of impact on pregnancy management and outcome. BMC Pregnancy Childbirth.

[6] (2019). World Health Organization. Obesity and overweight. https://www.who.int/news-room/fact-sheets/detail/obesity-and-overweight.

[7] (2019). National Health and Morbidity Survey (NHMS) 2019 Malaysia. Non-communicable diseases, healthcare demand, and health literacy. https://iptk.moh.gov.my/images/technical_report/2020/4_Infographic_Booklet_NHMS_2019_-_English.pdf.

[8] Kominiarek MA (2011). Preparing for and managing a pregnancy after bariatric surgery. Semin Perinatol.

[9] Heusschen L, Krabbendam I, van der Velde JM (2021). A matter of timing—pregnancy after bariatric surgery. Obes Surg.

[10] Snoek KM, Steegers-Theunissen RP, Hazebroek EJ, Willemsen SP, Galjaard S, Laven JS, Schoenmakers S (2021). The effects of bariatric surgery on periconception maternal health: a systematic review and meta-analysis. Hum Reprod Update.

[11] Sheiner E, Edri A, Balaban E, Levi I, Aricha-Tamir B (2011). Pregnancy outcome of patients who conceive during or after the first year following bariatric surgery. Am J Obstet Gynecol.

[12] Tack J, Arts J, Caenepeel P, De Wulf D, Bisschops R (2009). Pathophysiology, diagnosis and management of postoperative dumping syndrome. Nat Rev Gastroenterol Hepatol.

[13] Dave DM, Clarke KO, Manicone JA, Kopelan AM, Saber AA (2019). Internal hernias in pregnant females with Roux-en-Y gastric bypass: a systematic review. Surg Obes Relat Dis.

